# Estimating the Effect of Adhering to the Recommendations of the 2019 Canada’s Food Guide on Health Outcomes in Older Adults: Protocol for a Target Trial Emulation

**DOI:** 10.2196/65182

**Published:** 2025-01-23

**Authors:** Didier Brassard, Nancy Presse, Stéphanie Chevalier

**Affiliations:** 1 School of Human Nutrition, McGill University Sainte-Anne-de-Bellevue, QC Canada; 2 Faculty of Medicine and Health Sciences, University of Sherbrooke Sherbrooke, QC Canada; 3 Research Institute of the McGill University Health Centre, McGill University Montreal, QC Canada

**Keywords:** aged, Canada’s Food Guide, diet, dietary guidelines, target trial emulation, hypothetical trial, Healthy Eating Food Index-2019, HEFI-2019

## Abstract

**Background:**

The 2019 Canada’s Food Guide provides universal recommendations to individuals aged ≥2 years. However, the extent to which these recommendations are appropriate for older adults is unknown. Although ideal, conducting a large randomized controlled trial is unrealistic in the short term. An alternative is the target trial emulation framework for causal inference, a novel approach to improve the analysis of observational data.

**Objective:**

This study aims to describe the protocol for a target trial emulation in older adults, with an emphasis on key aspects of a hypothetical sustained diet and physical activity intervention.

**Methods:**

To emulate the target trial, nonexperimental data from the Quebec Longitudinal Study on Nutrition and Successful Aging (NuAge; N=1753 adults aged ≥67 years) will be used. NuAge includes 4 yearly measurements of dietary intakes, covariates, and outcomes. The per-protocol causal contrast will be the primary causal contrast of interest to account for nonadherence. The sustained intervention strategy will be modeled using the parametric g-formula. In the hypothetical trial, participants will be instructed to meet sex-specific minimal intakes for vegetables and fruits, whole grains, animal- and plant-based protein foods, milk and plant-based beverages, and unsaturated fats. The eligibility criteria, follow-up, intervention, outcomes, and causal contrast in the emulation will closely align with those of the target trial, with only minor modifications. We will attempt to emulate the randomization of treatment by adjusting for baseline covariates and prebaseline dietary habits.

**Results:**

Data collection for NuAge was completed in June 2008. For this study, the main analysis was started in May 2024. Submission of the manuscript is expected by February 2025.

**Conclusions:**

Emulating a target trial will provide the first evidence of the adequacy of the 2019 Canada’s Food Guide recommendations for older adults in relation to health outcomes.

**International Registered Report Identifier (IRRID):**

DERR1-10.2196/65182

## Introduction

### Background

The latest edition of Canada’s Food Guide (CFG) was published in 2019 [1]. Compared with the previous edition, key changes include the removal of the prespecified number of servings to consume each day, a shift toward qualitative (eg, “eat plenty of...”) instead of quantitative recommendations, and the provision of universal recommendations instead of age- and sex-specific recommendations. In addition, CFG recommendations primarily aim to reduce chronic disease risk. The evidence supporting CFG recommendations focuses on reducing the risk of cardiovascular disease, cancer, and type 2 diabetes risk [2]. However, evidence from a nationally representative survey of adults aged ≥65 years from Canada suggested that greater adherence to recommendations was insufficient to meet calcium, vitamin D, and folate requirements [3]. In Canada, one-third of community-dwelling older adults are at high nutrition risk [4,5], highlighting the importance of maintaining adequate nutritional status in this stratum of the population. Similarly, in the absence of specific recommendations on the amount of protein foods to eat, older adults may be eating less protein than required to maintain muscle mass [6-8]. CFG also provides brief physical activity recommendations but without explicit acknowledgment of the importance of these recommendations for older adults [1]. Indeed, performing a minimal amount of physical activity is paramount to maintaining muscle mass [6,7]. Thus, the universal recommendations in CFG may not be appropriate for older adults because they face unique challenges in consuming a healthy diet [9] and may require specific nutritional strategies [10].

Ideally, a randomized controlled trial (RCT) should be conducted to investigate the adequacy of CFG recommendations in older adults. However, such an RCT is unlikely to be conducted in the short term. An alternative is the target trial emulation framework for causal effect estimation using observational data [11-14]. Informally, the target trial emulation framework aims to emphasize and resolve design challenges in observational data analysis by explicitly emulating a hypothetical trial to estimate a causal effect [11]. In nutritional epidemiology, common issues with design and analyses can yield results that are largely inconsistent with those of randomized trials [15]. For example, the lack of consideration of the compositional nature of diet can dramatically influence effect estimates, that is, increasing the intake of one food must be compensated by decreasing the intake of another food in substitution modeling [15-17]. Furthermore, diet is a lifelong sustained exposure. In an observational study, the effect of diet assessed at a given time may actually reflect the cumulative exposure to previous dietary habits. In turn, ignoring previous dietary habits may result in a misalignment of “time zero” [18], as dietary habits are not randomly assigned at the beginning of the observational study. In other words, ignoring previous dietary habits makes it impossible to distinguish the effect of prospective or hypothetical dietary modification from the effect of retrospective dietary habits. The target trial framework is a helpful tool to highlight and address common issues in nutritional epidemiology. Ultimately, a successful emulation of the target trial based on observational data could yield effect estimates that more closely align with those of a hypothetical future RCT. Example applications of the target trial framework include the emulation of interventions on diet [19-22], physical activity [23], or both [24].

### Objectives

To the best of our knowledge, a target trial framework has not been used to assess the effect of adhering to CFG recommendations. Accordingly, this study aims, first, to describe the protocol for the emulation of a target trial using observational data from the Quebec Longitudinal Study on Nutrition and Successful Aging (NuAge [25]), which includes a cohort of 1753 adults aged 67 to 84 years at baseline, and, second, to address key aspects of the target trial emulation in the context of a sustained lifestyle intervention strategy involving diet and physical activity. Key aspects are the description of the sustained lifestyle intervention strategy, the attempt to emulate randomization, as well as assumptions and limitations specific to diet intervention. Notably, more general introductory texts to the target trial framework are available elsewhere [11,12,14].

## Methods

### Research Question and Hypothesis

Explicitly acknowledging the causal nature of a research question is a prerequisite to causal effect estimation using observational data [26-28]. This study aims to examine the adequacy of the universal dietary recommendations provided by CFG for older adults. Expressed as a counterfactual statement, we aim to answer the following question: What would be the difference in a given health outcome at the end of the follow-up if all eligible participants had increased their adherence to CFG recommendations on healthy food choices, compared to if they had maintained their habitual diet?

Specifically, among adults aged 67 to 84 years followed for 3 years, and compared with maintenance of habits, we aim to (1) estimate the causal effect of adhering to CFG dietary recommendations on markers of muscle health (eg, physical function and muscle strength), general health (eg, waist circumference, blood pressure, and glucose), and cognitive health (ie, Modified Mini-Mental State Examination) and (2) estimate the causal effect of adhering to a *reformulation* of CFG dietary recommendations, including more protein foods and a minimal physical activity recommendation, to amplify the positive health effects.

Accordingly, we hypothesize the following:

- *Hypothesis 1: adhering to recommendations positively influences general and cognitive health but does not influence muscle health.*
- *Hypothesis 2: increasing the consumption of protein-rich foods positively influences muscle health, and meeting minimal physical activity recommendations (≥30 min per day) further amplifies positive health effects.*

### Study Design and Sample

Data from the NuAge prospective cohort will be used to emulate the target trial [25]. The NuAge cohort comprised 1753 generally healthy community-dwelling adults aged 67 to 84 years at baseline and followed for 3 years. The baseline and each annual follow-up evaluation included a comprehensive assessment of sociodemographic data, diet, physical activity, functional status, as well as physical and mental health status [25].

The NuAge sample is relevant to our research question. The target population of CFG recommendations comprises all individuals aged ≥2 years, which is compatible with the NuAge target sample of generally healthy older adults from the greater Montreal, Sherbrooke, and Laval areas of the province of Quebec, Canada.

### Target Trial

The target trial framework has been suggested as a potential solution to improve the analysis of nutritional epidemiology studies aiming at causal inference [13]. Informally, the target trial framework helps to align the observational data analysis with that of a hypothetical trial. This framework is appropriate for the research question in this study, as we aim to estimate the effect of adhering to a hypothetical diet and physical activity intervention using observational data. The first step of a target trial emulation is the description of the target trial, that is, the protocol for the hypothetical RCT we wish to conduct [11,14]. The second step is the emulation, that is, describing how the target trial is emulated and conducting the study described in this protocol.

Table 1 presents the target trial and its emulation using observational data from NuAge. Key differences between the target trial and its emulation are that participants will be required to provide complete dietary assessment and covariate data at baseline (ie, eligibility component) and that we will attempt to emulate the randomized assignment by adjusting for dietary intakes before baseline as well as baseline covariates (ie, assignment component). Each part of the target trial and its emulation are described in the subsequent sections.

**Table 1:** Emulation of a dietary intervention target trial using observational data from the Quebec Longitudinal Study on Nutrition and Successful Aging (NuAge [25]).

Trial component	Target trial specification	Target trial emulation
Eligibility criteria	NuAge inclusion criteria: individuals aged 67-84 years; living in Montreal, Laval, or Sherbrooke; not cognitively impaired; and free of disabilities in activities of daily living Exclusion criteria: class II heart failure, chronic obstructive pulmonary disease requiring home oxygen therapy or oral steroids, inflammatory digestive diseases, and cancer treatment in the past 5 years	The inclusion and exclusion criteria are the same as those specified in the target trial. Furthermore, participants will be required to have complete baseline dietary assessment (at least one 24-hour recall with ≥500 kcal and FFQ^a^) and provide baseline covariate data.
Interventions^b^	Each individual would be assigned to 1 of the 4 following strategies: Control group (habitual diet, ie, typical North American diet) Adherence to Canada’s Food Guide recommendations on healthy food choices Adherence to Canada’s Food Guide recommendations on healthy food choices and including a high-protein reformulation Adherence to Canada’s Food Guide recommendations on healthy food choices, including a high-protein reformulation and a minimal physical activity component Each strategy is followed until the end of follow-up. Participants assigned to a lifestyle strategy are expected to maintain their dietary intake or amount of physical activity at or above the prespecified threshold by the corresponding intervention strategy.	The intervention component will be the same as that specified in the target trial. Furthermore, we will assume that each dietary assessment period (ie, within 2 months beginning at each time point) accurately reflects the average diet in the interval between follow-ups.
Assignment	Participants are randomly assigned to a dietary strategy but are not blinded to their assignment.	We will attempt to emulate randomized assignment by adjusting for dietary intakes before baseline and baseline covariates.
Outcomes	The outcomes are physical function and muscle strength, general health indicators, and cognitive health.	The outcomes will be physical function and muscle strength, general health indicators, and cognitive health.
Time zero and follow-up	The study starts at baseline and ends at incomplete follow-up or 3 years after baseline, whichever occurs first.	The study will start at baseline and end at incomplete follow-up or 3 years after baseline, whichever occurs first. An incomplete follow-up is defined as missing data for questionnaires (nonresponse or loss to follow-up) or missing outcome data at the end of follow-up.
Causal contrast^c^	Intention-to-treat effect Per-protocol effect	Observational analog of both contrasts: Secondary: intention-to-treat effect Primary: per-protocol effect
Statistical analysis	Intention-to-treat analysis: apply inverse probability weighting with adjustment for prebaseline and baseline factors associated with incomplete follow-up to account for study dropouts Per-protocol analysis: apply the parametric g-formula algorithm to compare postintervention outcomes between groups receiving each treatment strategy, adjusting for pre- and postbaseline factors associated with adherence to intervention strategies and incomplete follow-up.	Analysis will be the same as that specified in the target trial for both contrasts. However, the observational analog will require additional adjustments for confounding at baseline and before baseline due to previous dietary pattern.

^a^FFQ: food-frequency questionnaire.

^b^Refer to the *Hypothetical Interventions* section for detailed intervention.

^c^The observational analog of the intention-to-treat contrast corresponds to the baseline values of the intervention, which are assigned and initiated at the same time.

### Eligibility Criteria

The inclusion criteria for the target trial are the same as those for NuAge [25]. In the emulation, participants will be required to have at least one 24-hour dietary recall completed at baseline with ≥500 calories, as well as complete covariate data at baseline, as identified in the *Assignment* section.

### Hypothetical Interventions

#### Overview

The hypothetical intervention strategies evaluated will be as follows:

1. No change in dietary habits or physical activity (similar to a control intervention)
2. Adherence to CFG recommendations
3. Adherence to CFG recommendations, including reformulation (ie, higher intake of protein foods)
4. Adherence to CFG recommendations, including reformulation (ie, higher intake of protein foods) and performing at least 30 minutes of aerobic physical activity

Physical activity recommendations are not traditionally at the forefront of CFG recommendations. However, CFG does mention that “at least 150 minutes of moderate-to vigorous-intensity aerobic physical activity per week ... is recommended to achieve health benefits” [1]. Thus, recognizing the key role of exercise in maintaining health and muscle for older adults, the fourth hypothetical intervention includes a formal physical activity recommendation. In the target trial, the physical activity corresponds to performing aerobic exercise of light to vigorous intensity for at least 30 minutes per day [6].

#### The Challenge of a Well-Defined Nutritional Intervention

Emulating a well-defined dietary intervention for CFG recommendations is challenging. First, recommendations in the latest edition of CFG are qualitative and flexible (eg, “eat plenty of vegetables and fruits” [1,29]). Thus, various suitable yet distinct dietary patterns can align with the recommendations. Second, CFG recommendations target both food intakes (eg, vegetables and fruits) and nutrients (eg, saturated fats). The nutrient-based recommendations can be met by modifying the consumption of various foods. For example, to achieve the hypothetical intervention of “decreasing consumption of calories from saturated fats,” one could decrease saturated fats from dairy, nuts, and low nutritive value foods altogether. Arguably, the relationship between these food categories and a given health outcome may vary greatly.

To estimate a causal effect using observational data, the hypothetical interventions must be clearly defined to the point where “no meaningful variation” in the intervention remains [30,31]. In other words, the hypothetical diet interventions should be elaborated until no additional dietary characteristics are deemed impactful regarding the outcome of interest. Another consideration is that the modeling of hypothetical interventions should ideally be conducted with dietary intakes expressed using the same units. For example, mixing food intakes expressed in servings and grams in a statistical model may cause poor estimation of causal effects [17]. Finally, the statistical approach used to account for total energy or total food intake also affects the causal effect of interest and should be consistent with the research question [17,31-33].

#### Diet Simulations

For this study, adherence to CFG recommendations was defined based on simulated diets generated by Health Canada [34] and summarized in Multimedia Appendix 1 [34]. The simulated diets were designed to meet CFG recommendations on healthy food choices and nutrient requirements (Dietary Reference Intake).

These diets achieve near-perfect Healthy Eating Food Index (HEFI)-2019 scores (>78/80) through relatively high intake of recommended foods (ie, vegetables and fruits, whole grain foods, protein foods, and unsweetened milk and plant-based beverages with protein) and null intakes of foods not recommended (ie, non–whole grain foods; other low nutritive value foods; juice, sugary drinks and alcohol; and fatty foods rich in saturated fats). The HEFI-2019 score indicates the extent to which dietary intakes are consistent with CFG recommendations on healthy food choices [29,35].

Notably, the HEFI-2019 could have been used as a main exposure to measure adherence to CFG. However, the use of a composite score metric would not completely satisfy the criterion of a well-defined intervention to estimate a causal effect. First, high HEFI-2019 scores and high adherence to CFG recommendations can be achieved through many different strategies or dietary patterns. In the context of observational data, the specific strategies through which individuals achieve a high HEFI-2019 score would be based on dietary habits and patterns self-selected by the participants. This approach is similar to asking hypothetical trial participants to modify their intakes without clearly specifying how, which would obscure the estimated causal effect. Second, the HEFI-2019 score includes recommendations on foods and nutrients. As described earlier, mixing servings and grams in statistical models may cause poor estimation of causal effects [17].

We stress that the diets simulated by Health Canada were not actually consumed by older adults. Therefore, the simulated values for vegetables and fruits, whole grain foods, and plant-based protein foods *exceed* the 99th percentile of the distribution of usual intakes of these food categories, as estimated in adults aged ≥65 years from the Canadian Community Health Survey 2015–Nutrition [3]. In Table 2, the target intakes for vegetables and fruits, whole grains, and plant-based protein foods in the adherence to the 2019 CFG recommendations intervention were revised to correspond, at most, to the 90th percentile of the distribution of usual intakes among Canadians aged ≥65 years in 2015 [3].

**Table 2:** Emulation^a^ of hypothetical diet and exercise interventions by sex in the Quebec Longitudinal Study on Nutrition and Successful Aging (NuAge) cohort [25].

Sex		Recommended foods (RA^b^/d)							Dietary supplement^c^		Foods and beverages not recommended (RA/d)^d^					Physical activity (min/d)^e^
		Vegetables and fruits	Whole grains	Plant-based protein foods	Animal-based protein foods	Milk and plant-based beverages with protein	Unsaturated oils and fats			Other foods		Sugary drinks and alcohol	Non–whole grains			
**Control (no change)^f^**																
	Male individuals	—^g^	—	—	—	—	—	—		—		—	—		—	
	Female individuals	—	—	—	—	—	—	—		—		—	—		—	
**Adhering to the 2019 Canada's Food Guide recommendations on healthy food choices^h^**																
	Male individuals	6	1.5	1.0	2.0	1.0	1	No change		Minimum		Minimum	Minimum		No change	
	Female individuals	5	1.5	0.8	1.5	1.0	1	No change		Minimum		Minimum	Minimum		No change	
**Adhering to the 2019 Canada's Food Guide recommendations on healthy food choices, including extra protein^i^**																
	Male individuals	6	1.5	1.5	3.5	1.5	1	No change		Minimum		Minimum	Minimum		No change	
	Female individuals	5	1.5	1.3	3.0	1.5	1	No change		Minimum		Minimum	Minimum		No change	
**Adhering to the 2019 Canada's Food Guide recommendations on healthy food choices, including extra protein and physical activity**																
	Male individuals	6	1.5	1.5	3.5	1.5	1	No change		Minimum		Minimum	Minimum		30 or more	
	Female individuals	5	1.5	1.3	3.0	1.5	1	No change		Minimum		Minimum	Minimum		30 or more	

^a^The emulation of all hypothetical interventions will be implemented using a substitution approach in statistical models. In all models, 1 variable for total food intake and 1 variable for total beverage intake will be included, and foods not recommended will be left out from the models (ie, non–whole grain foods; other low nutritive value foods; juice, sugary drinks and alcohol; and fatty foods rich in saturated fats).

^b^RA: reference amount.

^c^Dietary supplements were not intervened on but were, nonetheless, excluded from foods and beverages not recommended to avoid being considered in the substitution. In other words, participants would not be instructed to modify their dietary supplements in the hypothetical trial.

^d^Minimum indicates that consumption would be set at the smallest amount, permitting a concomitant increase in recommended foods to meet Canada’s Food Guide targets. Portions for foods not recommended may vary on an individual basis.

^e^Physical activity corresponds to aerobic exercise of moderate or higher intensity [6].

^f^Values will be the averages observed at baseline in the NuAge cohort. In other words, values will be the observed intakes for the food categories or amount of physical activity when no change is applied.

^g^Not applicable.

^h^Values are derived from Health Canada’s simulated composite diets of adults aged ≥71 years. Participants would be expected to meet these targets for each food category. The specific food choices within these categories would be at the participants’ discretion. Values for vegetables and fruits, whole grain foods, and plant-based protein foods were truncated to correspond, at most, to the 90th percentile of the distribution of usual intakes among Canadians aged 65 years in 2015.

^i^Extra protein foods were added as follows: +0.5 RA of plant-based protein foods (eg, 25 g of nuts), +1.5 RA of animal-based protein foods (eg, 150 g of cooked unprocessed red meat, fish, or poultry or 3 small eggs), +0.5 RA of milk or plant-based beverage with protein (eg, 125 mL of milk or plant-based beverages with sufficient protein).

Because the 2019 CFG does not have a portion size system, reference amounts (RAs) were used as a proxy for servings. RAs are regulated quantities of foods that reflect the portion size typically consumed at 1 sitting in Canada. RAs were used by Health Canada to simulate a diet consistent with the 2019 CFG recommendations and dietary reference intake (Multimedia Appendix 1 [34]); therefore, RAs are adequate for this study.

#### Implementation

In the target trial, the sustained intervention strategy could be implemented as follows:

- Step 1: the participants’ usual dietary intake and physical activity would be assessed by research dietitians at each study visit.
- Step 2: if reported food intakes and duration of physical activity were equal to or above the prespecified thresholds (Table 2), no change would be suggested to the participants’ diet or physical activity. If food intakes or duration of physical activity were below the prespecified thresholds, participants would be instructed to increase food consumption to exactly the prespecified portions or increase physical activity duration to 30 minutes per day (when applicable).
- Step 3: if changes are required, participants would be instructed to decrease consumption of foods not recommended by the same amount as the increase in step 2. For example, if a 2-serving increase in vegetables and fruits is required to meet the prespecified intervention thresholds, participants would be instructed to substitute 2 servings of vegetables and fruits for non–whole grain foods; other low nutritive value foods; juice, sugary drinks and alcohol; and fatty foods rich in saturated fats.

In the emulation, for all hypothetical interventions, substitution will be implemented by including total intakes as a covariate and excluding foods not recommended from the models. More precisely, a variable reflecting total food intake (in RA/d) and a variable reflecting total beverage intake (in RA/d) will be included in all models. Hence, total food and beverage intakes will be constant across hypothetical diet interventions. In this approach to account for total energy, all model coefficients reflect the action of increasing intakes of recommended foods and a concomitant decrease in *any* of the foods not recommended [32]. On one hand, this approach can be potentially confusing [17,33], as the default interpretation of model coefficients assumes increasing the intake of each *food included in the model* while simultaneously decreasing the intake of foods *excluded from the model* [32]. On the other hand, the standard model is generally consistent with the implementation of dietary intervention in feeding trials [16,36,37]. The standard model also reduces the number of variables to be considered as intervention variables. Otherwise, 4 additional dietary components would have to be modeled for foods not recommended (ie, non–whole grain foods; other low nutritive value foods; juice, sugary drinks and alcohol; and fatty foods rich in saturated fats). Finally, the explicit description of the intervention strategies in the target trial protocol clarifies the estimand of interest, as done in an earlier study [19,31].

Notably, nutrient-based recommendations in CFG (ie, saturated fats, free sugars, and sodium intake) are not explicitly modeled to avoid the problems associated with mixed-unit models [17]. In the target trial, we assume that nutrient-based targets would be met by reducing consumption of foods not recommended (ie, non–whole grain foods; other low nutritive value foods; juice, sugary drinks, and alcohol; and fatty foods rich in saturated fats). In that regard, food-level substitution analyses in Canadians support this assumption for saturated fats [38,39].

The extra protein intervention consists of increasing the intake of both animal-based and plant-based protein foods by 1.5 and 0.5 RA per day, respectively, as well as milk and plant-based beverages with sufficient protein by 0.5 RA per day. Regarding the amount of food, this corresponds to adding 150 g of cooked unprocessed red meat, fish, or poultry or 3 small eggs; 25 g of nuts and seeds; and 125 mL of milk or plant-based beverages with sufficient protein while proportionally decreasing the intake of foods not recommended. In a previous RCT [40], older women aged 60 to 90 years were able to consume an additional 160 g of cooked lean red meat without substitution, thereby supporting the feasibility of the protein intervention in this hypothetical study.

### Assignment

**Figure 1:**
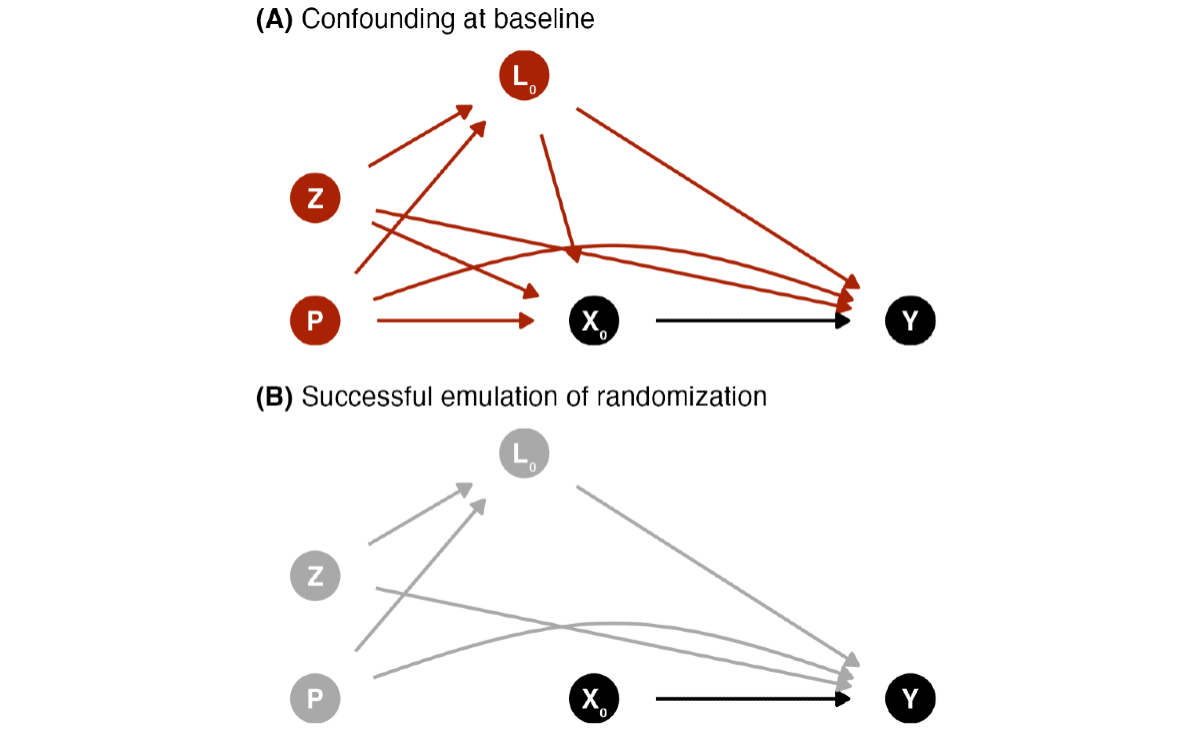
Causal directed acyclic graph (DAG) depicting (A) confounding and (B) successful emulation of randomization using g-methods at baseline between the intervention strategy (X) and outcome (Y). Baseline covariates (both time-invariant [Z] and time-varying [L]) and previous diet and physical activity habits (P) must be considered to emulate randomization. Time-varying treatment and covariates are not shown in this DAG to focus on randomization emulation. Subscripts indicate the time points, where 0 represents baseline.

We will attempt to emulate random allocation or randomization by adjusting for dietary intakes in the year before the intervention, as well as adjusting for covariates at the start of the study. Covariates were identified using the causal diagrams depicted in Figure 1, based on background knowledge of the relationship between the hypothetical lifestyle intervention and outcomes.

Dietary components that are the foundation for healthy eating in CFG include intakes of vegetables and fruits, whole grains, protein foods (plant- and animal-based protein foods, milk, and plant-based beverages with protein), and unsaturated oils and fats [1].

Covariates, including age at baseline, biological sex, region, education, living alone, smoking and drinking (alcohol) habits, major chronic diseases (ie, hypertension, diabetes, cancer, and heart disease), the number of medications, supplement use (eg, vitamins and minerals), and height and weight, are as follows:

1. *Z*, baseline covariates: age, sex, region, education, history of smoking, height, and former cancer history
2. *P*, previous exposure (ie, exposure of time-varying intervention before baseline): dietary habits before baseline
3. *L*, (time-varying) covariates: weight, number of medications, supplement use, living alone, major chronic diseases (eg, hypertension, diabetes, cancer, and heart disease), and smoking and alcohol habits
4. *X*, (time-varying) treatment: diet and physical activity habits
5. *Y*, end of follow-up outcome: muscle health, general health, and cognitive health

Contrary to dietary habits, data on physical activity habits before baseline were not collected in NuAge. In this case, the potential effect of previous physical activity habits will not be accounted for in the models that aim to emulate the sustained physical activity intervention strategy. For the models emulating the sustained diet intervention strategy only, physical activity habits during the study will be used as a covariate, hence mitigating the confounding of previous physical activity, at least to some extent.

A successful emulation of randomization requires that there is no unmeasured confounding. However, this is never guaranteed with observational data. Thus, we emphasize our assumptions that (1) the causal graph accurately depicts the relationship under study and (2) the covariates included are a sufficient set of covariates to address confounding.

### Outcomes

The primary outcomes will be the mean end of follow-up values for muscle strength (ie, handgrip using vigorimeter, elbow flexor, and knee extensor) and physical function (ie, normal and fast walking and “timed up-and-go”). Outcome values were measured according to a standardized protocol in NuAge [25].

For secondary outcomes, mean end of follow-up values for a set of relevant variables will be considered by domains:

- *General health:* waist circumference, blood pressure (systolic and diastolic), blood glucose, and estimated glomerular filtration rate
- *Cognition:* the modified Mini-Mental State Examination score

### Time Zero and Follow-Up

In the target trial of a sustained lifestyle intervention, participants would be met at baseline and then regularly to ensure that diet and physical activity habits are consistent with the intervention assigned by the random allocation. The hypothetical diet and physical activity intervention would be assigned and initiated at baseline. In the emulation, annual follow-ups with comprehensive diet, physical activity, and covariate data collection are available to emulate the hypothetical intervention. Hence, participants would be followed from the study baseline (time point 0; ie, the time at which the intervention strategy would also be assigned and would begin), at each year (ie, time points 1 and 2), and until the end of the study (time point 3). We also assume that the diet and physical activity habits measured at each follow-up time adequately reflect the habits during the entire year.

The end of follow-up outcome measurements will be used to estimate the effect of the sustained lifestyle intervention strategy. Measures of dietary intake and physical activity throughout the study (ie, time point 0 to time point 3) will be used to emulate the sustained lifestyle intervention. Dietary intakes in the year preceding the intervention will be estimated using the frequency questionnaire completed at baseline (ie, time point 0). Missing covariate data at a given follow-up will be carried forward once, after which participants will be considered as having incomplete follow-up.

### Causal Contrast

The estimand of interest in this study, the target causal effect of a sustained lifestyle intervention strategy, is

*E*(*Y*^1,1,1,1^|*C* = 0) – *E*(*Y*^0,0,0,0^|*C* = 0) **(1)**

that is, the expected value of a given health outcome *Y* at the end of follow-up if all participants had increased their adherence to CFG recommendations on healthy food choices and physical activity, when applicable, at all 4 time points (*X_k_=1*, *always intervene*) versus if all participants had maintained their habitual diet and physical activity (*X_k_=0*, *never intervene*). The estimand (equation 1) also indicates that all participants completed the intervention (C=0), that is, in the absence of incomplete follow-up.

The causal contrasts of interest are the observational analogs of intention-to-treat and per-protocol analyses [11]. Given the observational design, participants are not expected to have followed a treatment strategy unknown to them at the time of data collection. Therefore, the primary analysis will be the per-protocol contrast of a sustained lifestyle intervention strategy. In the per-protocol analysis, nonadherence to the hypothetical interventions can be accounted for. In the target trial, participants with a condition after baseline that would have prevented or limited participation in a hypothetical lifestyle intervention would be allowed to discontinue the intervention (eg, lengthy hospitalization, prolonged bed rest, and incident cancer). In the emulation, if such conditions occur in a sufficiently large number of participants, these participants will be “excused” from following the hypothetical intervention [23]. In other words, participants who would have been unable to pursue the study due to major events will not be considered as having incomplete follow-up if they attended the annual assessment. Allowing participants to discontinue adhering to the hypothetical intervention strategy mitigates confounding by the disease burden [23].

The intention-to-treat analysis will be a secondary analysis of a hypothetical point intervention, for example, dietary counseling at baseline only. Notably, it will not be possible to conduct an intention-to-treat analysis identical to that of a controlled study where the interest is to estimate the effect of being *assigned* to an intervention [11]. However, it is possible to conduct an observational analog of the intention-to-treat analysis. In the observational analog, the intention-to-treat analysis aims to estimate the impact of a hypothetical intervention in which adherence is measured at baseline only.

### Statistical Analysis

#### Modeling of Hypothetical interventions

Stratification and multivariable regression (ie, covariate adjustment) are conventional statistical approaches to address confounding in nutritional epidemiology. However, the conventional approaches are not adequate to estimate cumulative treatment effects (eg, diet over time) in the presence of time-varying confounding (eg, weight status over time) and treatment (eg, previous diet) [41,42]. In this study, nonadherence to the hypothetical interventions and incomplete follow-up will be considered using general g-methods for the per-protocol analysis [11,42]. Among g-methods, the parametric g-formula provides the most flexibility for analyses involving hypothetical dietary interventions, as used in previous studies [19,22,43]. Briefly, in the context of an observational study, the parametric g-formula and its implementation into an R package (R Foundation for Statistical Computing) [44] use parametric models to predict the joint history of previous diet and physical activity habits (ie, the hypothetical sustained intervention strategy) and confounding variables. For example, linear regression models are used to predict continuous covariates (eg, body weight), while logistic regressions are used to predict binary or categorical variables (eg, indicator variable for dietary supplement use). The per-protocol causal contrast of the hypothetical intervention presented in Table 2 is then emulated based on Monte Carlo simulated data generated using the g-formula algorithm [44]. The parametric g-formula correctly accounts for time-varying confounding in the presence of feedback between the intervention and the confounding variables, as confounding is addressed using standardization [41,42]. Furthermore, standardization allows for estimating an average causal effect (ie, marginal effect) consistent with the estimand of interest (equation 1) rather than a conditional effect. In summary, “threshold interventions” that depend on the reported dietary intakes or amount of physical activity [43,45] and the parametric g-formula [44,46] will be used to emulate the intervention of *consuming at least x servings of food* and *doing at least x minutes of light to vigorous physical activity.*

**Figure 2:**
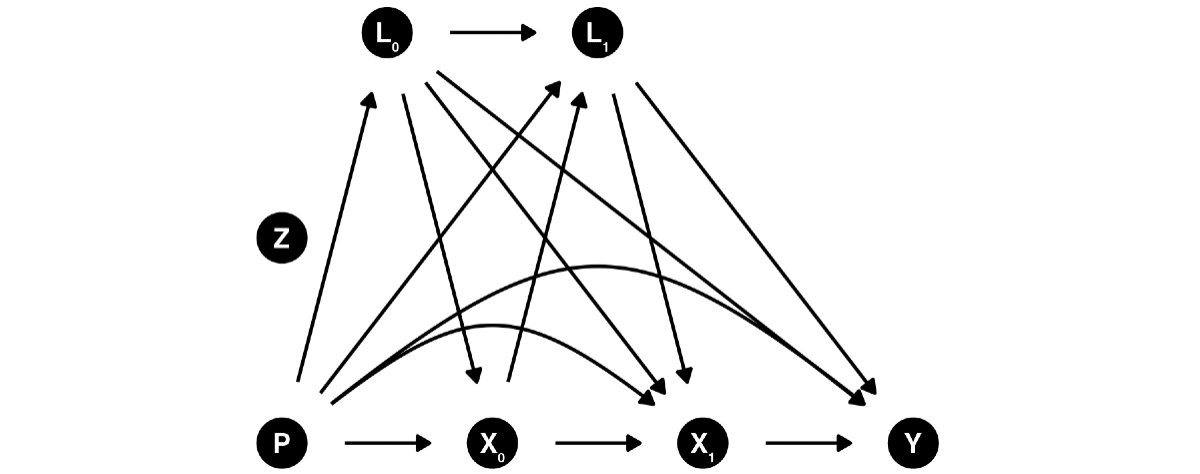
Directed acyclic graph depicting the hypothesized relationship among previous dietary exposure, time-varying interventions (diet and physical activity), and covariates. Arrows from the Z node are not shown for clarity but would point toward all baseline and time-varying nodes, as well as the outcome. Only 1 follow-up is shown for visualization purposes, but the hypothesized causal structure extends to additional follow-ups. Subscripts indicate the time points, where 0 represents baseline and 1 represents time point 1.

Figure 2 presents the causal directed acyclic graph (DAG) of the hypothesized relationship between a sustained lifestyle intervention strategy (*X_0_, X_1_*) and an end of follow-up outcome *Y* for 1 follow-up after baseline (year 1). The model is limited to year 1 for clarity, but the hypothesized causal structure extends to additional follow-ups. The exposure of interest *X_k_* is the joint and cumulative effect of a sustained diet and physical activity intervention strategy measured at baseline and follow-ups.

In the context of this target trial emulation (Figure 2), *P* includes dietary habits before the baseline assessment. *P* can have an effect on baseline dietary habits (eg, previous healthy habits increase the likelihood of baseline healthy habits) and dietary habits throughout the target trial emulation (eg, previous healthy habits increase the likelihood of adhering to healthy habits). Furthermore, *P* influences baseline and time-varying confounding. Finally, given the long-term effect of chronic exposure, *P* potentially affects *Y* directly.

The intention-to-treat analysis is similar to the per-protocol analysis. However, only baseline diet and physical activity habits and covariates are considered, as well as prebaseline diet and physical activity. In both the per-protocol and intention-to-treat analyses, loss to follow-up (eg, nonresponse or missing follow-up and health outcomes not measured) will be accounted for using g-methods such as inverse probability weighting.

#### Dietary Assessment

In NuAge, diet during the year before baseline was assessed using 1 semiquantitative food-frequency questionnaire [47]. Dietary intakes at baseline (ie, time point 0) and each annual follow-up (ie, time points 1, 2, and 3) were assessed using 3 repeated face-to-face interviewer-administered 24-hour dietary recalls.

Dietary intakes measured using 24-hour dietary recall are more accurate (ie, have less systematic error or bias) than food-frequency questionnaire [48-50] but are particularly affected by random measurement error (ie, within-individual random error) [50]. When several variables measured with errors are considered simultaneously in a regression model, the regression coefficients may be biased in any direction [51]. To account for random measurement error, the National Cancer Institute Markov Chain Monte Carlo (NCI MCMC) multivariate method could be applied [52]. However, the combination of the parametric g-formula and multivariate measurement error correction using the NCI MCMC method is not feasible. The NCI MCMC method estimates time-invariant measurement error–corrected intakes, while the g-formula algorithm is designed for time-varying exposures.

Recognizing the importance of accounting for measurement error, the correction for measurement error will be reserved for the secondary intention-to-treat analysis. For the intention-to-treat contrast, the time-varying values of exposure and the time-varying values of confounding are not considered. Thus, the NCI MCMC method will be used to obtain measurement error–corrected estimates of the relationship between dietary intakes measured at baseline and outcome at the end of follow-up. Notably, the three 24-hour dietary recalls collected at each time point contribute to reducing random errors, at least to some extent, even in the absence of measurement error correction.

#### Sensitivity Analysis to Assess the Impact of Measurement Error

For the secondary intention-to-treat analysis, results based on the measurement error–corrected and –uncorrected dietary intakes will be compared. The difference between the estimated relationships will allow to extrapolate the impact of not accounting for random errors in the primary *per-protocol* analysis.

#### Physical Activity Assessment

Physical activity throughout the study will be assessed using the Physical Activity Scale for the Elderly questionnaire [53,54]. Rather than the total Physical Activity Scale for the Elderly score, specific questions estimating the total time of physical activities will be used to be consistent with the intervention strategy.

#### Covariates and Subgroups

To the extent permitted by the number of observations for each outcome, continuous covariates will be modeled using restricted cubic splines with 3 to 5 knots (at percentiles 10-50-90, 5-35-65-95, or 5-27.5-50-77.5-95) [55]. Categorical covariates will be modeled to ensure a sufficient sample size at each level.

The effect of dietary changes will be estimated for the entire sample. The sample will also be stratified by biological sex to reflect both potential biological differences and, to some extent, gender differences (although not reported).

#### Variance Estimation

The variance will be estimated using a minimum of 200 bootstrap sample replicates to consider uncertainty at each step of the estimation [56].

#### Software and Code

The main statistical analyses will be conducted using R software (version 4.3.1 or greater) and the *gfoRmula* package [44,57]. The manuscript results will be generated using Quarto markdown (Posit PBC). Codes for main analyses and generation of manuscript results will be shared in a publicly available code repository.

### Ethical Considerations

The original NuAge protocol was approved by the research ethics boards of the *Institut universitaire de gériatrie de Montréal* and the *Institut universitaire de gériatrie de Sherbrooke* (Quebec, Canada). The NuAge Database and Biobank [58] has received approval by the *Centre intégré universitaire de santé et de services sociaux de l’Estrie—Centre hospitalier universitaire de Sherbrooke* Research Ethics Board. Secondary analyses of data from the NuAge Database and Biobank for the study described in this protocol are approved by the McGill University Research Ethics Board Office (#22-11-041).

All participants of NuAge provided informed consent. From the initial cohort of 1793 participants, 1753 (97.77%) agreed to the integration of their data and biological samples into the NuAge Database and Biobank for future studies.

Secondary analyses based on the NuAge Database and Biobank use deidentified data, which do not allow participants to be identified by the investigators.

NuAge participants voluntarily consented to participate and were not provided with monetary compensation.

## Results

Data collection for NuAge was completed in June 2008. For this study, the main analysis based on the final curated data started in May 2024. The manuscript will be written according to the Strengthening the Reporting of Observational Studies in Epidemiology statement. We anticipate the submission of the manuscript to a peer-reviewed academic journal by February 2025.

## Discussion

### Principal Findings

In this study protocol, we have described a target trial to assess the effect of adhering to CFG recommendations on healthy food choices. The emulation will be performed using data from the NuAge Database and Biobank [58]. Benefiting from the flexibility of observational data, we also aim to compare adherence to multiple reformulations of CFG recommendations, including the effect of increasing the intake of protein-rich foods and the amount of aerobic physical activity on selected health outcomes. Furthermore, we have outlined the rationale for using simulated diets to emulate adherence to CFG recommendations, the process of selecting covariates to attempt to emulate randomization with causal diagrams, and the challenges of addressing random measurement error.

We emphasize that the purpose of emulating a target trial using observational data is to improve the quality of observational analysis [11,14]. In other words, the target trial framework aims to support the coherence between the causal research question and the observational data analysis [26]. However, estimating causal effects with nonexperimental observational data depends on strong assumptions. The key assumptions are that there are no unmeasured confounders, no measurement errors, and no model misspecifications (eg, functional form of covariates and model outcome distribution) [19]. We first recognize that the absence of residual or unmeasured confounding cannot be guaranteed. The extent to which this assumption is sufficiently satisfied depends on the appraisal of covariates considered. In that regard, we have used graphical tools, DAG, to explicitly describe our analytical assumptions and to identify confounding variables [59-61]. Second, the absence of measurement error assumption will not be satisfied considering the use of dietary intake data measured with 24-hour dietary recalls. On one hand, 24-hour dietary recalls have the least systematic error or bias compared with other common instruments, such as food-frequency questionnaires [48,49]. On the other hand, 24-hour dietary recalls are largely affected by within-individual random errors [50], which can cause bias in any direction in multivariable models [51], as in this study. This issue is mitigated, at least to some extent, by using average data from 3 repeated 24-hour dietary recalls at each follow-up. Sensitivity analyses comparing estimates based on measurement error–corrected and –uncorrected baseline dietary intakes will be used to assess the impact of random measurement errors. Third, the absence of model misspecification will be assessed by examining differences between the observed value of time-varying covariates and the predicted value of time-varying covariates as modeled with the g-formula.

### Limitations

The strengths of this study and protocol include the explicit emulation of a hypothetical trial, the thorough description of the emulation of the sustained dietary intervention, and the use of background knowledge and DAG to derive a sufficient set of confounders. Limitations must be addressed. First, the NuAge sample size is relatively limited (N=1753), although comprehensive nutrition and covariate data were collected. Second, the target food intakes based on diet simulations from Health Canada *exceeded* the 99th percentile of the usual intake distribution of Canadians aged ≥65 years from Canada in 2015 [3]. A revision of the dietary intervention targets may be needed if observed dietary intakes in NuAge deviate significantly from targets (Table 2), as was done in a previous nutrition target trial emulation [22]. Third, the presence of random measurement error associated with 24-hour dietary recalls may bias estimates. Finally, the target trial emulation cannot replace an actual RCT. Evidence from an RCT will be required to confirm the value of either CFG recommendations or the enhanced CFG recommendations.

### Conclusions

In conclusion, the target trial framework is useful for estimating the causal effect of adhering to CFG recommendations using nonexperimental data when an RCT is impractical [11,19]. Coupled with key assumptions, including the absence of unmeasured confounding, the absence of measurement error, and no model misspecification, we believe that the emulation will provide timely evidence regarding the effect of adhering to CFG recommendations in older adults and inform on the added value of a reformulation.

## Appendix

Multimedia Appendix 1 Healthy Eating Food Index-2019 dietary constituents and score among simulated diets by Health Canada, by age and sex group.

## Abbreviations

CFG: Canada’s Food Guide
DAG: directed acyclic graph
HEFI: Healthy Eating Food Index
NCI MCMC: National Cancer Institute Markov Chain Monte Carlo
NuAge: Quebec Longitudinal Study on Nutrition and Successful Aging
RA: reference amount
RCT: randomized controlled trial

## Data Availability

The datasets analyzed during this study are available in the GitHub repository [62].
